# A Case of Spontaneous Intestinal Perforation in Osteogenesis Imperfecta

**DOI:** 10.4021/jocmr369w

**Published:** 2010-08-18

**Authors:** Katherine Wheatley, Ee Ling Heng, Mary Sheppard, Hank Schneider, Neil Moat, Jeremy Cordingley, Sundeep Kaul

**Affiliations:** a Royal Brompton Hospital, London, UK; b Chelsea and Westminster Hospital, London, UK

## Abstract

A 51-year-old male with known osteogenesis imperfecta (OI) (type 1) presented with symptoms and signs of infective endocarditis. Transthoracic echocardiography showed chordal rupture and free mitral regurgitation, resulting in an emergency mitral valve repair. The surgical procedure was largely uneventful but subsequent clinical course on the intensive care unit was complicated by bowel perforation requiring two laparatomies for a colonic resection and loop ileostomy formation. Histology of the excised tissue demonstrated absent musculature with no evidence of ischemia. Spontaneous non-ischemic bowel perforation as a complication of osteogenesis imperfecta is to date unreported. Our case highlights the need for a high index of suspicion of non-ischemic bowel perforation in patients with connective tissue disorders.

**Keywords:**

Osteogenesis imperfecta; Bowel perforation; Collagen; Non-ischemic; Connective tissue disorders; Pathogenesis; Collagen vascular disorder; Acute abdomen

## Case Report

A 51-year-old male patient presented to his local hospital with sudden onset atypical chest pain and dyspnoea on exertion. He had no previous history of cardio-pulmonary disease but had positive risk factors for ischemic heart disease namely hypertension and hypercholesterolemia. However, he was known to have a diagnosis of osteogenesis imperfecta with a significant past medical history of multiple childhood fractures. There was also a strong family history of osteogenesis imperfecta with multiple affected siblings.

**Figure 1.**
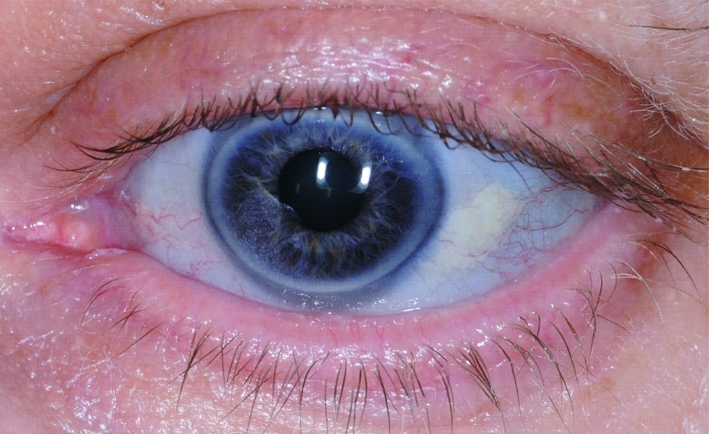
Blue sclera of patient.

Clinical examination revealed the well recognised extra skeletal features of OI namely blue sclera (Fig. 1) and ligamentous laxity. Tachypnoea with a respiratory rate of twenty-six was noted. Auscultation of the praecordium revealed a grade IV pansystolic mumur heard loudest in the mitral area - previously unrecorded and assumed to be new in onset. There were no peripheral stigmata of endocarditis and no evidence of fluid overload. An electrocardiogram (ECG) showed sinus rhythm with no signs of acute ischemia. The presence of pyrexia, likely new-onset murmur and raised inflammatory markers led to a working diagnosis of infective endocarditis. An empirical antibiotic regimen of intravenous benzylpenicillin and gentamicin was commenced after serial blood cultures were taken. An urgent transthoracic echocardiogram demonstrated a bright mobile mass attached to the anterior mitral valve leaflet (AVML) suggestive of vegetation with moderate to severe mitral regurgitation.

Over the next 48 hours, the patient developed pulmonary oedema, initially requiring intravenous diuretic therapy and non-invasive ventilation followed by invasive ventilatory and inotropic support. He was then transferred to our tertiary cardiothoracic unit for emergency surgery. A three-dimensional echocardiogram performed on transfer revealed free, torrential mitral regurgitation with chordae rupture. No vegetations were reported. A bicuspid aortic valve with mild eccentric aortic regurgitation was also noted. An intra-aortic balloon pump was sited prior to surgery. There was no electrocardiographic or laboratory evidence of acute or cardiac ischemia.

Intra-operatively, ruptured chordae of the AMVL was found and mitral valve (MV) ring annuloplasty was performed. At the time of the operation the surgeon noted that the tissue was extremely friable. A lengthy post-operative intensive care course followed, with hemofiltration for metabolic acidosis secondary to transient renal impairment. Due to probable systemic inflammatory response syndrome (SIRS), inotropes and vasopressors were required to maintain satisfactory hemodynamic parameters and a slow ventilatory wean ensued.

On day 12 post MV repair, the patient developed sudden onset severe abdominal pain. Clinically, he was noted to have an acute abdomen with peritonism. He had been hemodynamically stable for at least one week post-operatively. Plain abdominal and chest radiographs demonstrated dilated bowel loops and free air under the diaphragm. An emergency laparotomy revealed caecal perforation with dilated ascending colon and subsequent fecal contamination. There was no evidence of mechanical obstruction. The gastrointestinal surgeon noted exceptionally indurated and friable tissue intra-operatively, to the extent that the normal bowel manipulation to form an ileostomy led to splitting of the mesentery, subsequent bleeding and handling-induced ischemia of the distal bowel segment. A technically challenging right hemicolectomy due to poor tissue integrity was performed with primary anastamosis. A planned second-look laparotomy was undertaken the next day, which necessitated a loop ileostomy formation due to the development of a gangrenous distal ileum.

**Figure 2.**
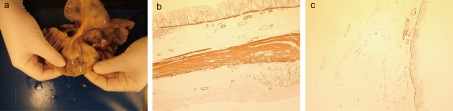
(a) Macroscopic specimen of perforated bowel; (b) Transverse section of large bowel immunostained for smooth muscle actin (SMA) with positive brown staining for SMA in muscularis mucosae, thick layer of circular and longitudinal muscle in area away from the area of perforation; (c) Transverse section of caecal large bowel from patient showing preserved muscularis mucosa muscle with total absence of the circular and longitudinal muscle fibres in the wall.

The main histological findings of the excised tissue revealed a complete absence of circular and longitudinal muscle fibres with preservation of the muscularis mucosa of the perforated caecal segment (Fig. 2). Interestingly no histological signs of concurrent ischemia were present with an absence of mucosal oedema, hemorrhage, vascular congestion, necrosis or dusky discolouration [1]. The changes were focal within the caecum with other areas of the large bowel possessing normal layers of muscle (Fig. 2). In the context of the surgical findings of extremely friable tissue, it was concluded that rupture was not due to bowel ischemia but rather an intrinsic connective tissue defect with absence of the muscle layers in the caecum.

The patient was finally discharged with good clinical recovery after a period of rehabilitation.

## Discussion

The case highlights the occurrence of non-ischemic bowel perforation in a patient with osteogenesis imperfecta and the importance of maintaining a high index of suspicion in this patient group.

Osteogenesis Imperfecta (OI) is predominantly an autosomal dominant disorder of Type I collagen with variable penetrance. It occurs in approximately 1 in 10,000 births [2], with four recognised subtypes based on disease severity and progression [3]. The manifestations are multi-systemic, with the majority being skeletal and cardiac complications. Disease severity is proportional to the extent of abnormal type I collagen [4]. Patients present with fractures relating to increased bone fragility and low bone mass. Other extraskeletal features include blue sclera, dentinogenesis imperfecta, ligamental laxity and hearing impairment [5]. Cardiac pathologies tend to involve left sided valves with aortic regurgitation most commonly, followed by mitral regurgitation [4]. Valvular dysfunction occurs infrequently in osteogenesis imperfecta, with only 4 of 109 subjects screened by Hortop et al demonstrating valve pathology [6].

Limited literature is available for gastrointestinal associations of osteogenesis imperfecta. A PubMed, Embase and Medline literature search with key words ‘osteogenesis imperfecta’, ‘gastrointestinal’, ‘perforation’ and ‘cardiovascular’ was applied. This revealed two case reports of severe megacolon and large bowel obstruction secondary to pelvic deformities [7]. A case series of 43 patients with osteogenesis imperfecta Type III by Lee et al documents higher incidences of recurrent abdominal pain, chronic constipation and fecal impaction in these patients [8]. These manifestations are often related to acetabular protrusion causing pelvic narrowing. Other potential gastrointestinal complications in osteogenesis imperfecta include mesenteric ischemia, gastrointestinal bleeds and ulceration, pancreatitis, cholecystitis, perforation, and liver failure [9]. Mesenteric ischemia occurs in less than 0.2-0.4% of all cases [10], but has an associated mortality of 70-100% [9, 11].

We are not aware of any case reports of spontaneous gut perforation associated with osteogenesis imperfecta, whereas it has been highlighted in one case report as a complication associated with Ehlers-Danlos syndrome (EDS) [12]. Osteogenesis and EDS are both commonly of autosomal dominant inheritance and caused by collagen defects. EDS affects collagen types I and V. Osteogenesis imperfecta classically involves gene mutations in collagen type I (COL1A1 and COL1A2) which overlaps with the gene mutations encoding Ehlers-Danlos. Therefore it is reasonable to postulate that the underlying collagen defect in this patient has significantly contributed to the perforation.

Our patient required an extended period of intensive care with inotropic and vasopressor support post bypass surgery. This is associated with an increased risk of ischemic bowel injury, yet both the intra-operative and histological findings are consistent with a non-ischemic cause of bowel perforation. A mesenteric angiogram would have added further evidence to support the lack of ischemia but abdominal radiographs revealed dilated bowel loops and perforation obviating the need for further imaging and leading to urgent surgical intervention.

This case highlights the necessity to consider a rare but potentially life-threatening complication of osteogenesis imperfecta. Median survival is dependent upon the subtype, with no significant increased mortality in patients with type IA OI [13]. The link between non-ischemic bowel perforation and OI has not been described before and there is very limited data on the gastrointestinal associations of osteogenesis imperfecta.

We propose that the pathogenesis of the rupture in this particular patient was due to the defective collagen in his bowel wall, which had already received a subclinical insult from a drop in cardiac output following cardiac surgery. We conclude it is imperative to maintain a high index of suspicion for the signs and symptoms of bowel perforation in this patient population. We recognize that there have been no other similar cases published to date and that data from further case series are required to evaluate the importance of gastrointestinal complications in patients with osteogenesis imperfecta.
